# Cytotoxicity of Tumor Antigen Specific Human T Cells Is Unimpaired by Arginine Depletion

**DOI:** 10.1371/journal.pone.0063521

**Published:** 2013-05-23

**Authors:** Markus Munder, Melanie Engelhardt, Diana Knies, Sergej Medenhoff, Guido Wabnitz, Claudia Luckner-Minden, Nadja Feldmeyer, Ralf-Holger Voss, Pascale Kropf, Ingrid Müller, Roland Conradi, Yvonne Samstag, Matthias Theobald, Anthony D. Ho, Hartmut Goldschmidt, Michael Hundemer

**Affiliations:** 1 Third Department of Medicine (Hematology, Oncology, and Pneumology), University Medical Center Mainz, Mainz, Germany; 2 Department of Internal Medicine V, University of Heidelberg, Heidelberg, Germany; 3 Institute of Immunology, University of Heidelberg, Heidelberg, Germany; 4 Department of Immunology and Infection, London School of Hygiene and Tropical Medicine, London, United Kingdom; 5 Department of Medicine, Section of Immunology, Imperial College London, London, United Kingdom; 6 Transfusion Center, University Medical Center Mainz, Mainz, Germany; 7 National Center for Tumor Diseases, University of Heidelberg, Heidelberg, Germany; Maisonneuve-Rosemont Hospital, Canada

## Abstract

Tumor-growth is often associated with the expansion of myeloid derived suppressor cells that lead to local or systemic arginine depletion via the enzyme arginase. It is generally assumed that this arginine deficiency induces a global shut-down of T cell activation with ensuing tumor immune escape. While the impact of arginine depletion on polyclonal T cell proliferation and cytokine secretion is well documented, its influence on chemotaxis, cytotoxicity and antigen specific activation of human T cells has not been demonstrated so far. We show here that chemotaxis and early calcium signaling of human T cells are unimpaired in the absence of arginine. We then analyzed CD8^+^ T cell activation in a tumor peptide as well as a viral peptide antigen specific system: (i) CD8^+^ T cells with specificity against the MART-1_aa26–35*A27L_ tumor antigen expanded with *in vitro* generated dendritic cells, and (ii) clonal CMV pp65_aa495–503_ specific T cells and T cells retrovirally transduced with a CMV pp65_aa495–503_ specific T cell receptor were analyzed. Our data demonstrate that human CD8^+^ T cell antigen specific cytotoxicity and perforin secretion are completely preserved in the absence of arginine, while antigen specific proliferation as well as IFN-γ and granzyme B secretion are severely compromised. These novel results highlight the complexity of antigen specific T cell activation and demonstrate that human T cells can preserve important activation-induced effector functions in the context of arginine deficiency.

## Introduction

The fate of a growing tumor is not only based on the proliferative capacity of the cancer cell itself but rather dictated by the complex interplay of various invading cell types, most prominently antitumoral and regulatory immune cells. The endogenous or therapy-induced antitumoral immune attack is often inhibited by tumor immune escape mechanisms [1], [2]. Among these, so-called myeloid-derived suppressor cells (MDSC) inhibit effectively antitumoral adaptive immune responses mainly by the production of reactive oxygen intermediates and by the expression of the arginine-metabolizing enzymes nitric oxide synthase and arginase [3], [4]. Two mammalian arginase isoforms exist, which both hydrolyze arginine to ornithine and urea [5]. The isoforms differ with respect to cellular and subcellular expression and regulation. Murine and human MDSC have been shown to express the hepatic isoform arginase I constitutively or inducibly [6]. Arginase I-mediated arginine depletion in the tumor microenvironment leads to inhibition of T lymphocyte proliferation, cytokine synthesis and anti-tumor immune responses [6], [7]. In human T lymphocytes, the absence of arginine induces a downregulation of the signal transducing T cell receptor-associated ζ chain [8], [9], impairs dephosphorylation of the actin-binding protein cofilin [10] and inhibits progression through the cell cycle via induction of a G0–G1 arrest [11].

The defect of the adaptive immune system due to arginase-mediated arginine depletion is causally responsible for the unrestricted tumor growth in various murine tumor models [12] and human tumor entities [12], [13], [14]. Arginase inhibition or arginine substitution can reconstitute polyclonal human T cell reactivity [12] or induce tumor cell death [14] in primary material from human cancer patients *in vitro*.

The influence of arginine depletion on the antitumoral immune response of antigen-experienced human CD8^+^ T lymphocytes with defined tumor antigen specificity has not been demonstrated so far. For an effective antitumoral adaptive immune response, T cells have to migrate within the tumor microenvironment and – after recognition of tumor-associated cognate peptides and TCR-mediated activation – perform various effector functions (cytotoxic killing, secretion of cytokines) as well as initiate proliferation and differentiation to expand clone size and generate effector and memory cells. A detailed analysis of these various aspects of T cell activation is important for the situation in tumor patients *in vivo* upon antigen specific antitumoral vaccination and is especially relevant in light of the minimal success of protein-, peptide- or dendritic cell (DC)-based cancer vaccines [15]. Although the expansion of tumor specific T lymphocytes with *in vitro* antitumoral activity has been demonstrated in various vaccination protocols of patients, this does not translate into effective tumor regression [16], [17]. Clinical inefficiency correlates with the presence of functionally inactive tumor-infiltrating lymphocytes within the tumor stroma [14] whereas they can regain functional potential outside the tumor microenvironment [18], [19].

Among a variety of known tumor antigens, the superior T cell immunogenicity of the tumor antigen MART-1_aa26–35_ (melanoma-associated antigen recognized by T cells, amino acids 26–35) was demonstrated in numerous *in vitro* analyses [20] and also clinical trials [21]. Furthermore the MART-1_aa26–35*A27L_ analogue peptide, with a substitution of the amino acid alanine (A) by leucine (L) at position 27 showed superior immunogenicity [21]. While MART-1 is expressed quite selectively on malignant melanoma cells, there is also cross reactivity with multiple myeloma [22], bronchial [23] and renal cancer cells [24], due to a homologue peptide sequence between MART-1 and the HM1.24 antigen. Therefore, MART-1_aa26–35*A27L_ is an ideal model antigen in order to analyze tumor specific T cell responses covering numerous tumor entities.

We report here that human T cell chemotaxis, early calcium signaling and MART-1_aa26–35*A27L_ specific CD8^+^ T cell mediated cytotoxicity are uncompromised in the absence of arginine while interferon-gamma (IFN-γ) and granzyme B secretion are suppressed when tumor antigen specific T cells were restimulated with the cognate peptide under arginine-limiting conditions. We validate these results in an alternative CMV pp65_aa495–503_ peptide specific system with expanded CMV pp65_aa495–503_ specific T cells as well as T cells retrovirally transduced with a pp65_aa495–503_ specific T cell receptor (TCR).

## Materials and Methods

### Blood Samples and Ethics Statement

To analyze the activation of T cells, peripheral blood/buffy coats from healthy donors (HD) were used. All human studies were performed after obtaining written informed consent in accordance with the Declaration of Helsinki and were approved by the Landesaerztekammer Rhineland-Palatine Ethics Committee and the ethics committee of the Medical Faculty, University of Heidelberg according to the institutional guidelines. Data safety management was performed according to the data safety regulations of the University Hospitals Heidelberg and Mainz.

### Cell Culture Medium and Reagents

If not specified otherwise, cells were maintained in RPMI 1640 cell culture medium with penicillin/streptomycin and 2 mM L-glutamine (all from PAA Laboratories, Pasching, Austria), either with 10% heat inactivated fetal calf serum (FCS) for cell lines or 5% heat inactivated human serum (both from PAA Laboratories) for primary cells. Arginine-free RPMI 1640 cell culture medium was purchased from PromoCell (Heidelberg, Germany) and supplemented with penicillin/streptomycin, 2 mM L-glutamine, 10% heat inactivated dialyzed FCS (PAA Laboratories) and 10 µM MnCl_2_. Arginine-free cell culture medium was supplemented with L-arginine to obtain medium with arginine. Unless otherwise specified, reagents were purchased from Sigma (Sigma-Aldrich, Steinheim, Germany).

### Peptides

The MART-1_aa26–35*A27L_ peptide (ELAGIGILTV) and HLA-A2 restricted irrelevant control peptide (LLIIVILGV; as a control for unspecific, non-tumor antigen mediated T cell activation) were synthesized by the peptide synthesis facility of the German Cancer Research Center Heidelberg using standard procedures. CMV pp65_aa495–503_ peptide (NLVPMVATV) was purchased from Biosyntan, Berlin, Germany.

### Isolation of PBMC and T cells

Peripheral blood mononuclear cells (PBMC) were purified from peripheral blood/buffy coats by ficoll-hypaque density gradient purification (Biochrom, Berlin, Germany). Purified T cells were obtained from PBMC using a T cell Isolation Kit (Miltenyi Biotec, Bergisch Gladbach, Germany) according to the manufacturer’s protocol.

### Expansion of MART-1_aa26–35*A27L_ Specific T cells

PBMC from HLA-A*02^+^ HD (Institute of Immunology and IKTZ, University of Heidelberg, Heidelberg, Germany) were used to generate antigen specific T cells. Immature DC were obtained by culturing plastic adherent PBMC for 5 days with GM-CSF (800 U/ml, Sargramostim, Bayer, Seattle, WA, USA) and IL-4 (500 U/ml, R&D Systems, Minneapolis, MN, USA). Maturation of immature DC was then induced by culturing the cells for 2 days in the presence of TNF-α (10 ng/ml, R&D Systems), IL-6 (1000 U/ml, PromoCell) and prostaglandin E_2_ (1 µg/ml, Biomol/Enzo Lifesciences, Lörrach, Germany). Simultaneously, MART-1_aa26–35*A27L_ peptide (10 µg/ml) was added to load DC. Afterwards, autologous PBMC were incubated for 7 days together with mature DC in the presence of 5% human serum and IL-2 (50 IE/ml, Proleukin, Chiron GmbH, Munich, Germany) to expand MART-1_aa26–35*A27L_ specific T cells. In case of cytotoxicity assays, incubation was extended to 28 days and restimulations with MART-1_aa26–35*A27L_ peptide pulsed T2 cells were performed (along with culture medium and IL-2 renewal) on day 7, 14, and 21. T2 cells were pulsed by 2 h-incubation in serum-free medium with a peptide concentration of 10 µg/ml.

### Expansion of Human CMV pp65_aa495–503_ Specific T cells

PBMC were purified from buffy coats of HLA-A*0201^+^ as well as HCMV seropositive donors by ficoll-hypaque separation. The isolated cells underwent positive magnetic cell sorting (Miltenyi Biotec) to obtain a pure CD8^+^ T cell subset. The untouched CD8^−^ fraction was loaded with 1 µg/ml pp65_aa495–503_ peptide for 8 h at 37°C. Afterwards, the cells were irradiated with 3500 rad. The CD8^+^ T cells were then stimulated with the irradiated, pp65_aa495–503_-loaded cells in 10% human serum for initially 2 days. Starting on day 3, human IL-2 (20 IE/ml, Roche Diagnostics GmbH, Mannheim, Germany) and human IL-7 (5 ng/ml, R&D Systems) were additionally supplemented. After one or two pp65_aa495–503_ peptide specific restimulations as well as CMV pp65_aa495–503_ specific tetramer sorting by flow cytometry a purity of >95% was reached for pp65_aa495–503_ peptide specific CD8^+^ T cells. The pp65_aa495–503_ specific T cell clone T21 was kindly provided by Helga Bernhard (V. Medical Department, Medical Center Darmstadt, Germany) and propagation of the clone was done essentially as described [25].

### Transfection of Human T cells with CMV pp65_aa495–503_ Specific TCR

Retroviral transduction of the pp65_aa495–503_ specific TCR was performed as described elsewhere [26] with the use of Fugene (Roche Diagnostics) as transfection agent. The pp65_aa495–503_ specific TCRα (Vα 18.1) and TCRβ (Vβ 13.1) chains [27] were kindly provided by Mirjam Heemskerk (University Medical Center, Leiden, The Netherlands), cloned into a modified retroviral pBullet vector harbouring drug-selection cassettes so as to accomplish normalized TCR expression [28]. The site-directed mutagenesis was performed close to the CDR3α region and inside of the CDR3β region as described [29], leading to a novel pp65_aa495–503_ specific TCR. For transduction, the wild type pp65_aa 495–503_ specific TCR from M. Heemskerk, the novel TCR as well as the hybrid TCRs composed of the combined TCR chains of wild type and novel TCR chains were used. The T cells were expanded by weekly stimulation with anti–human CD3/CD28 beads (Invitrogen, Darmstadt, Germany) and human IL-2 (20 IU/ml, Roche Diagnostics).

### Chemotaxis Assay

T cells were incubated in medium (+/− arginine) for 24 h or 48 h. Subsequently, 200 µl of the corresponding medium was added to the lower compartment of a 96-well transwell plate (pore size 3 µm; Corning, Lowell, MA, USA) with or without 100 ng/ml SDF-1α (stromal cell-derived factor-1 alpha; R&D Systems, Wiesbaden-Nordenstadt, Germany). 5×10^4^ T cells in 75 µl of medium were transferred to the upper compartment of the transwell plate in triplicates. To determine maximum migration cells were pipetted to the lower compartment of the transwell plate. After incubating the cells for 4 h, 150 µl of the cell suspension was retained from each lower well compartment and mixed with a calibrated plastic bead solution for quantification of cells by flow cytometry. The percentage of migrated cells was calculated in relation to the detected cells in the maximum migration setting, which was set to 100%.

### Calcium Flux Determined by Flow Cytometry

To determine calcium flux of T cells after activation, 1×10^7^ T cells were incubated in the presence or absence of arginine over night. Cells were loaded in 1 ml of fresh medium (+/− arginine, respectively) with the calcium indicating dye Indo 1-AM (final concentration 2 µg/ml; Sigma-Aldrich) for 60 min at 37°C in the dark. Next, anti-CD3 antibody (OKT3, final concentration 20 ng/ml; kindly provided by Prof. Dr. Yvonne Samstag, Institute of Immunology, University of Heidelberg, Heidelberg, Germany) or isotype control IgG2a (BD Biosciences, Heidelberg, Germany) were added to the cells and incubated for additional 10 min at room temperature. Flow cytometric analysis (LSRII flow cytometer, BD Biosciences) was done as follows: after measurement of unstimulated cells for 3 min, cross-linking goat-anti-mouse-IgG+IgM antibody (final concentration 10 µg/ml, Dianova, Hamburg, Germany) was added and cells were analyzed for additional 3 min. As a positive control, cells were then activated by ionomycin (final concentration 1 µM; Merck, Darmstadt, Germany).

### IFN-γ ELISPOT and IFN-γ/Granzyme B/Perforin ELISA

CD8^+^ cells were purified from the MART-1_aa26–35*A27L_ activated and expanded T cell population using positive immunomagnetic cell sorting (MACS-system, Miltenyi Biotec). Purified CD8^+^ cells were then incubated with MART-1_aa26–35*A27L_ peptide or irrelevant peptide pulsed T2 cells (2 h incubation with 10 µg/ml peptide) as targets for 24 h in anti-IFN-γ antibody (Mabtech, Nacka Strand, Sweden) coated nitrocellulose-plates (Millipore, Schwalbach, Germany) and plate-bound IFN-γ was subsequently detected as described previously [22]. To analyze MART-1_aa26–35*A27L_ specific IFN-γ synthesis by CD8^+^ cells, a E:T ratio of 1∶5 was used.

The concentration of IFN-γ in the supernatant of T cell activation cultures was determined as previously published [9]. Briefly, supernatants of stimulation cultures were harvested and cytokine concentrations were measured by specific capture ELISA according to the manufacturer’s instructions (BD Biosciences). All stimulation conditions were performed at least in triplicates. Granzyme B and perforin concentrations in ELISPOT or cell culture supernatants were also determined by ELISA according to manufacturer’s instructions (Mabtech). ELISA microplates were purchased from Greiner Bio-One (Frickenhausen, Germany).

### Intracellular IFN-γ Analysis by Flow Cytometry

The expression of intracellular IFN-γ and different surface T cell markers was measured by flow cytometry. After activation of MART-1_aa26–35*A27L_ expanded PBMC with MART-1_aa26–35*A27L_ or irrelevant peptide loaded T2 cells for 20 h, 10 µg/ml brefeldin A (Biomol/Enzo Lifesciences) were added to the cell culture 4 h prior to staining. Then cells were fixed in 4% paraformaldehyde (J.T. Baker, Deventer, Holland) for 10 min at room temperature. After washing, the cells were permeabilized with saponin buffer (PBS with 0.5% BSA, 5% FCS, 0.1% saponin and 0.1% NaN_3_) for 10 min at room temperature. Cells were then incubated with fluorochrome-labeled antibodies against CD3, CD8, CD28 and IFN-γ (all from BD Biosciences) according to the manufacturer’s instructions. As controls cells were stained with the corresponding isotype antibodies. After a final washing with saponin buffer, cells were resuspended in 0.5% paraformaldehyde in PBS. Flow cytometry analysis was performed with a FACS Calibur flow cytometer (BD Biosciences).

### Cytotoxicity Assay ([^51^Cr]-Chromium Release Assay)

In case of the MART-1 antigen model, CD8^+^ cells were purified from the MART-1_aa26–35*A27L_ activated and expanded T cell population using negative immunomagnetic cell sorting (CD8^+^ T cell isolation Kit, Miltenyi Biotec) and preincubated in the respective medium (+/− arginine) for 24 h. The next day T2 cells were pulsed with MART-1_aa26–35*A27L_ or irrelevant peptide (10 µg/ml, 2 h-incubation in serum-free medium) and subsequently labeled with [^51^Cr]-Chromium (final concentration: 125 µCi/ml; Hartmann Analytic, Braunschweig, Germany) for another 2 h in medium with 5% human serum. Finally, 10^4^ labeled T2 cells were seeded out in 96-well round-bottom plates with effector cells in different ratios (E:T range 1∶1–20∶1). After an incubation period of 4 h (37°C, 5% CO_2_), 75 µl of each supernatant were harvested and analyzed using a γ-counter (Perkin Elmer, Boston, MA, USA). Spontaneous and maximal release of [^51^Cr]-Chromium were determined by analyzing supernatants of T2 cell cultures without effector cells either in presence of medium (spontaneous release) or 2% Igepal CA-630 (maximal release). Specific lysis was calculated as follows: [(test – spontaneous release)/(maximal release – spontaneous release)×100 = % specific lysis]. All experiments were performed in duplicates.

In the CMV specific model, K562-A2 cells were used as target cells, loaded with pp65_aa495–503_ peptide at 3 different concentrations (1 nM, 10 nM, 100 nM) and labeled with [^51^Cr]-Chromium for 1.5 h. After washing, target cells were seeded in a 96-well round bottom plate and effector cells were added. After an incubation period of 4 h, 80 µl supernatant were harvested. The spontaneous and maximal release was determined in the presence of either medium or 1% Triton X-100, respectively.

### Tetramer Analysis

MART-1_aa26–35*A27L_ specific T cells were quantified in the expanded T cell population by staining with PE-labeled tetrameric MHC complexes loaded with the peptide ELAGIGILTV (iTAG MHC Tetramer; Beckman Coulter, Fullerton, CA, USA) according to the manufacturer’s protocol. Additionally cells were stained with fluorochrome-labeled antibodies against CD3 and CD8 (BD Biosciences) according to the manufacturer’s instructions. Finally cells were resuspended in 0.5% paraformaldehyde in PBS and analyzed using a FACS Calibur flow cytometer (BD Biosciences).

### Proliferation Analysis

For proliferation analysis via [^3^H]-Thymidine incorporation, the cells were pulsed with 1 µCi/well [^3^H]-Thymidine (GE Healthcare, Buckinghamshire, U.K.) after 48 h and incubated for another 16 h. Cells were harvested on glass fiber filters using an automatic cell harvester and the incorporation of [^3^H]-Thymidine was measured in a microplate scintillation counter. The K562-A2 cells were irradiated with 3500 rad before the assay. For CFSE-based analysis, T cells were labeled with 0.5 µM CFSE (Invitrogen, Karlsruhe, Germany) for 15 minutes at 37°C, centrifuged, incubated another 30 min in prewarmed cell culture medium and subsequently used for cell activation assays. Cells were then analyzed by flow cytometry.

### Statistical Analysis

Differences between T cell activation with or without arginine were analyzed by student’s t-test using the Statistica for Windows software (StatSoft, Tulsa OK, USA). Chemotaxis assays were analyzed by One-way ANOVA with Tukey’s multiple comparisons test. Differences in the number of spots per well in the IFN-γ ELISPOT experiments between T2 cells loaded with MART-1_aa26–35*A27L_ compared to T2 cells loaded with an irrelevant peptide were also calculated by student’s t-test and ELISPOTs were defined as positive, if MART-1_aa26–35*A27L_ peptide activation achieved at least 10 spots more than control peptide and if the difference was significant (p<0.05).

## Results

### Arginine Deficiency does not Inhibit Chemotactic Movement and Early Calcium Signaling of Human T cells

**Figure 1:**
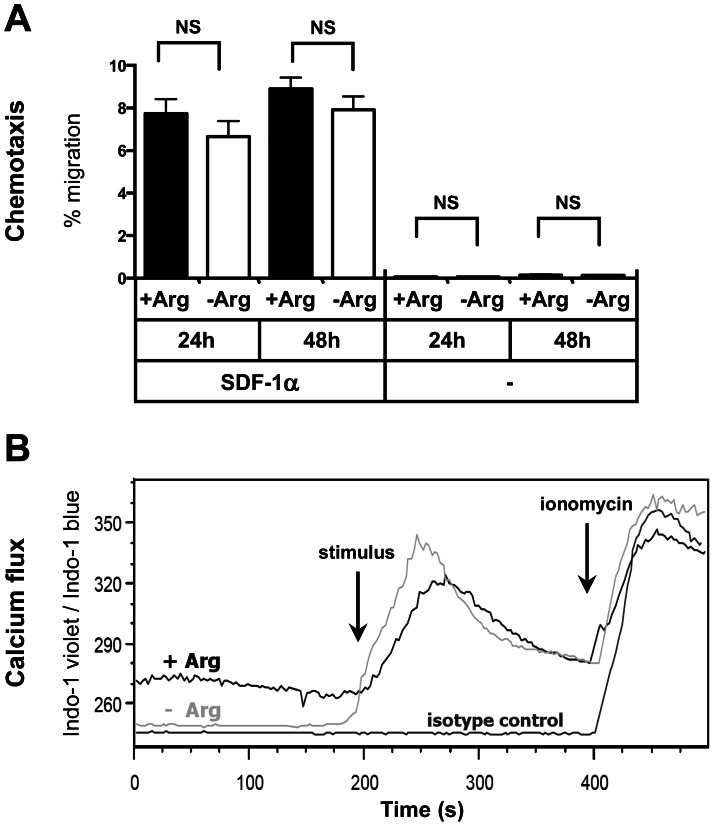
Arginine-independent T cell functions: chemotaxis and calcium flux. **A** Purified primary human T cells were preincubated either for 24 h or 48 h in the presence (+Arg, 1000 µM) or absence (−Arg, 0 µM) of arginine. They were then placed in the respective media into the upper compartment of a trans-well plate, whereas a chemokine (SDF-1α) was added to the lower compartment of the well. Wells without chemokine were also set up to detect spontaneous migration of cells. Maximum migration was determined by adding the same number of T cells to the lower compartment of a separate well. After 4 h cells in the lower compartment were quantified flow-cytometrically. Migration of T cells (%) was calculated relatively to the maximum migration which was set to 100%. Shown are summarized mean values and standard deviations of results from 3 individual experiments. NS: not significant. **B** Calcium signaling in primary human T cells in the presence (+ Arg, 1000 µM) or absence (−Arg) of arginine was analyzed by flow cytometry using the calcium indicating dye Indo 1-AM. The ratio of Indo-1 violet/Indo-1 blue correlates directly with the amount of calcium in the cell. T cells were preincubated with OKT-3 antibody and calcium levels of resting cells were monitored for 3 min. The cells were then stimulated by adding a crosslinking antibody ( = stimulus), which led to increasing calcium levels irrespective of arginine. As a negative control, isotype control antibody was used instead of OKT-3. Finally, the calcium ionophore ionomycin was added as a positive control. One representative analysis (total number of experiments = 3) is shown as a histogram plot overlay.

In order to analyze chemokine-induced migration of human T cells in the context of arginine deficiency we analyzed the amount of migrated T cells upon incubation with and without the chemokine SDF-1α in a transwell system in the absence or presence of arginine. Notably, the cells had been preincubated for either 24 h or 48 h already in the respective media in order to subject them to arginine deficiency for extended periods of time before the chemokine assay was started. As demonstrated in Fig. 1A, T cells were able to migrate along the SDF-1α chemokine gradient irrespective of arginine. Summarizing 3 individual experiments, 7.7% ±1.9% (24 h) or 8.9% ±0.9% (48 h) of the T cells migrated in the presence of arginine and 6.7% ±1.2% (24 h) or 7.9% ±1.1% (48 h) in the absence of arginine. There was no significant difference regarding T-cell migration between arginine-bearing or arginine-deficient conditions after 24 h (p = 0.71) or 48 h (p = 0.79). Spontaneous T cell migration was also not different: 0.07% ±0.03% (24 h) and 0.14% ±0.08% (48 h) with arginine vs. 0.07% ±0.03% (24 h) and 0.14% ±0.07% (48 h) without arginine (p>0.99 for both time points). Note that SDF-1α induced an up to 100 fold increase in the amount of migrated cells compared to spontaneous migration (p<0.0001 for each condition).

Next, we assessed activation-induced early calcium signaling in human T cells. We stimulated primary human T cells via their TCR and measured intracellular calcium flux flow-cytometrically (Fig. 1B). Again, T cells had been preincubated in the respective media overnight to mimic *in vivo* adaptation towards an arginine-deprived micromilieu. In 3 individual experiments we reproducibly saw that arginine deficiency did not impair TCR-triggered calcium flux in human T cells.

### Arginine Depletion Leads to Inhibition of IFN-γ and Granzyme B Secretion by Tumor Antigen Specific Human CD8^+^ T cells, Whereas Perforin Secretion is Unimpaired

Effective CD8^+^ T cell-mediated tumor control most likely depends on an unimpaired synergistic cooperation of various different T cell effector functions. Apart from activation-induced secretion of important effector cytokines like IFN-γ, T cells kill tumor cells via granzyme and perforin-mediated tumor-antigen specific cytotoxicity. We therefore analyzed the impact of arginine deficiency on secretion of IFN-γ/granzyme B/perforin and the cytotoxic capacity of T cells in the MART-1 tumor antigen specific model. MART-1_aa26–35*A27L_ antigen specific T cells from HLA-A*02^+^ HD were expanded by *in vitro* coincubation with MART-1_aa26–35*A27L_ pulsed donor-derived DC. Subsequently, the expanded CD8^+^ T cells were purified after 7 days and restimulated with T2 antigen presenting cells loaded with the MART-1_aa26–35*A27L_ peptide or an irrelevant control peptide in the presence/absence of arginine. IFN-γ secretion was then measured as marker of T cell activation, as it is clearly an important antitumoral effector function of CD8^+^ T lymphocytes and a commonly used read-out system for effective anti-tumor immunity [22]. This whole experimental approach was chosen to study if memory T cell activation in the context of antigen presenting cells and a defined prototypical tumor antigen recapitulates the activation impairment defined in the setting of antibody-mediated polyclonal or allogeneic stimulation [8].

**Figure 2:**
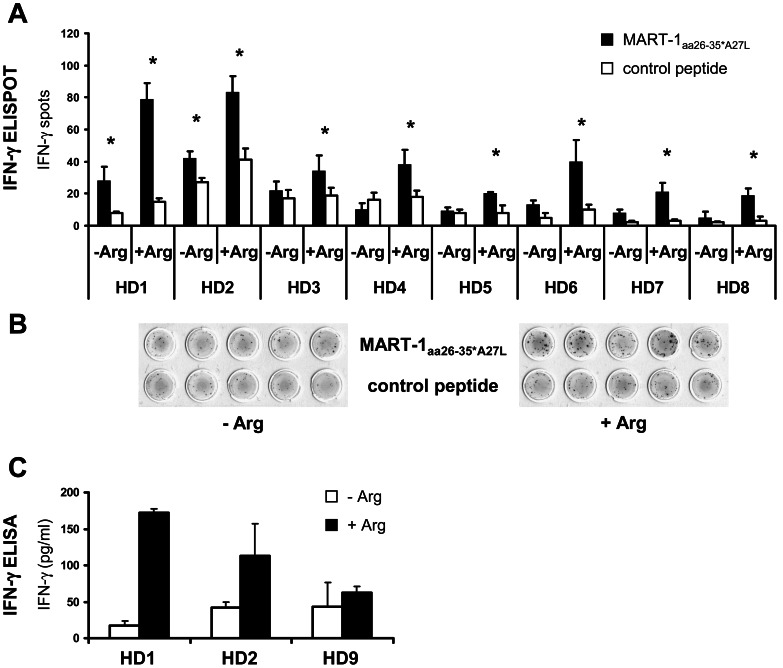
Impact of arginine depletion on activation of tumor antigen specific T cells analyzed by IFN-γ ELISPOT and IFN-γ ELISA. CD8^+^ MART-1_aa26–35*A27L_ specific T cells were expanded by activation with MART-1_aa26–35*A27L_ peptide loaded DC. CD8^+^ T cells were then restimulated with T2 cells loaded with MART-1_aa26–35*A27L_ peptide or control peptide for 24 h in ELISPOT assays in the presence of arginine (+Arg, 1000 µM) or in arginine-free cell culture medium (−Arg). **A** Results of all positive IFN-γ ELISPOT assays are presented: Black bars depict median numbers of IFN-γ spots for T cells incubated with MART-1_aa26–35*A27L_ peptide loaded T2 cells and white bars show stimulation with irrelevant control peptide. The asterisks (*) indicate a positive MART-1_aa26–35*A27L_ specific IFN-γ ELISPOT. Arginine depletion led in all HD to a significant reduction of IFN-γ spots (all individual p-values <0.05). All determinations were done at least in triplicate and each experiment was set up from a different HD (n = 8). **B** An exemplary IFN-γ ELISPOT assay (from HD 2) is demonstrated. **C** To quantify IFN-γ secretion, expanded MART-1_aa26–35*A27L_ specific CD8^+^ T cells were coincubated with MART-1_aa26–35*A27L_ peptide loaded T2 cells for 48 h either in the absence (−Arg) or presence of arginine (+Arg, 1000 µM). IFN-γ concentrations of the supernatants were determined by IFN-γ ELISA and are shown as mean of triplicates.

Of note, in 8 of 8 positive ELISPOT assays from individual donors IFN-γ secretion of T cells was significantly reduced (individual p-values for all HD <0.05) in the absence of arginine compared to the stimulation in the presence of arginine (Fig. 2A). When T cell activation with MART-1_aa26–35*A27L_ peptide loaded T2 cells in the presence of arginine is set as 100% and all 8 stimulation experiments are calculated together, median reduction of IFN-γ secretion is 63% ±13% (p<0.001). In 6 of 8 activation assays, the cytokine secretion was completely reduced to background level in the absence of arginine (as demonstrated by the absence of an asterisk in Fig. 2A). A representative ELISPOT assay is demonstrated in Fig. 2B.

To confirm these data, we quantified global IFN-γ secretion by ELISA in the supernatant of T cell activation cultures in the presence or absence of arginine. We detected measurable IFN-γ secretion in 3/3 ELISA assays. As demonstrated in Fig. 2C, we found significantly reduced IFN-γ secretion in our restimulation of MART-1_aa26–35*A27L_ antigen specific T cells in the absence of arginine. Summarizing the 3 experiments, there is a median reduction of 63% ±29% (p<0.05) in T cell IFN-γ secretion upon arginine depletion as compared to the stimulation in the presence of arginine.

**Figure 3:**
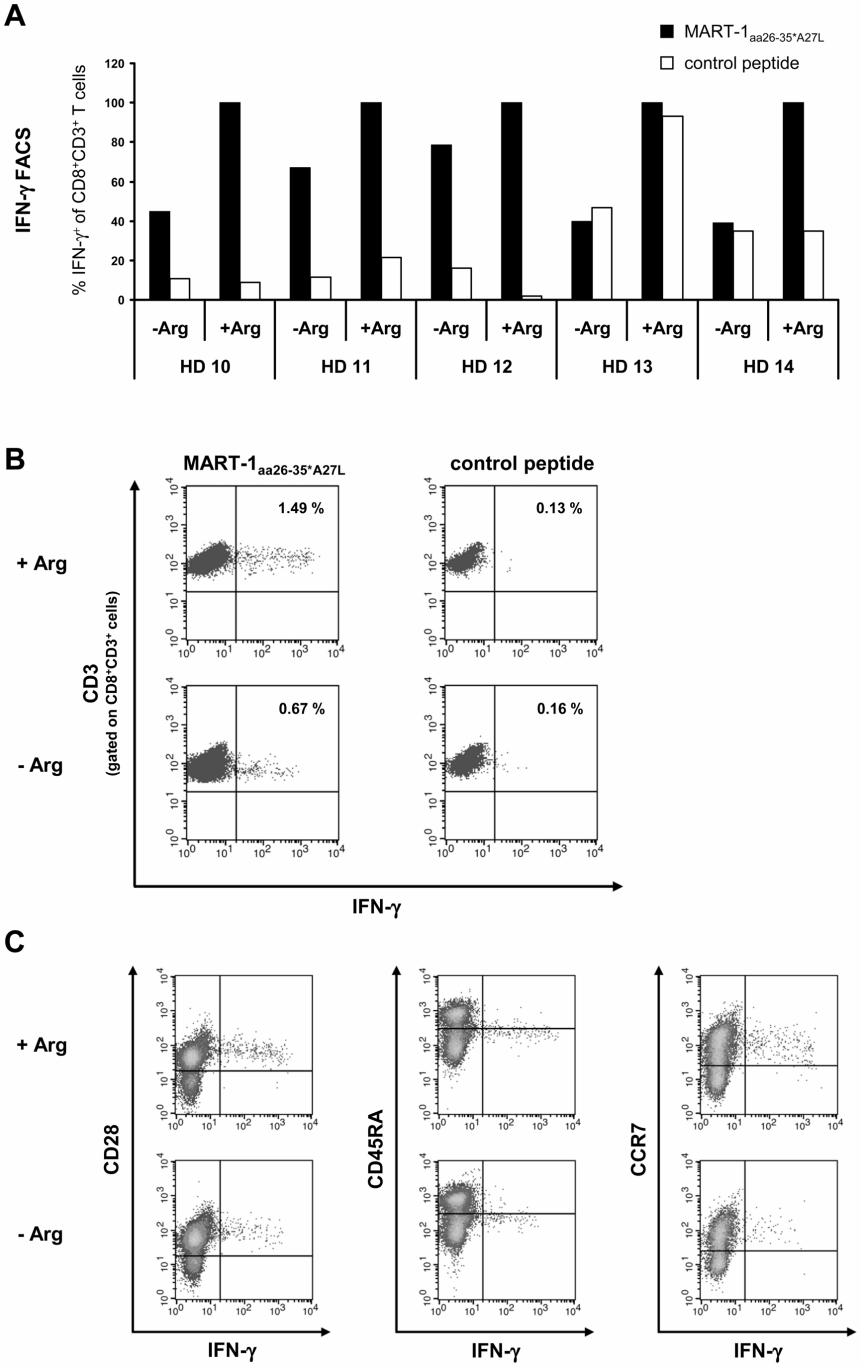
Impact of arginine depletion on activation of tumor antigen specific T cells analyzed by intracellular IFN -γ staining. CD8^+^ MART-1_aa26–35*A27L_ specific T cells of 5 HD were expanded and then restimulated by coincubation with MART-1_aa26–35*A27L_ (black bars) or irrelevant control peptide (white bars) loaded T2 cells either in the presence (+Arg, 1000 µM) or absence (−Arg) of arginine. After 24 h, cells were fixed, permeabilized and stained for intracellular IFN-γ as well as extracellular CD3, CD8, CD28, CD45RA and CCR7. After fixation cells were analyzed by flow cytometry. **A** The fractions of IFN-γ^+^ T cells are demonstrated relatively to the corresponding activation with MART-1_aa26–35*A27L_ peptide pulsed T2 cells in the presence of arginine, which was set as 100%. In all HD except of HD 12 the percentage of IFN-γ^+^ T cells was significantly reduced in the absence of arginine (p<0.05). **B** An exemplary intracellular IFN-γ flow cytometry analysis (from HD 10) is demonstrated. The numbers in the quadrants show the frequency of IFN-γ^+^ cells as a fraction of gated CD8^+^CD3^+^ lymphocytes. **C** IFN-γ^+^ CD8^+^CD3^+^ T cells express the CD28^+^ CD45RA^−/low^ CCR7^+^ phenotype of antigen experienced T cells. An exemplary flow cytometry analysis (from HD10) after activation with MART-1_aa26–35*A27L_ peptide loaded T2 cells is shown.

In addition, we analyzed MART-1_aa26–35*A27L_ specific T lymphocyte IFN-γ synthesis by intracellular flow cytometry. DC-expanded human MART-1_aa26–35*A27L_ specific T cells were restimulated with MART-1_aa26–35*A27L_ pulsed T2 cells in the presence or absence of arginine in 5 HD (Fig. 3). In all HD, the absence of arginine led to decreased amounts of IFN-γ producing CD8^+^ MART-1_aa26–35*A27L_ antigen specific T cells with a median reduction of 55% ±18%, p<0.001 (Fig. 3A and 3B). Of special interest, IFN-γ producing, MART-1_aa26–35*A27L_ specific CD8^+^ T cells expressed the CD28^+^ CD45RA^−/low^ CCR7^+^ phenotype of central memory T cells (Fig. 3C), representing antigen-experienced T cells after expansion and activation by MART-1_aa26–35*A27L_ pulsed DC and MART-1_aa26–35*A27L_ pulsed T2 cells.

**Figure 4:**
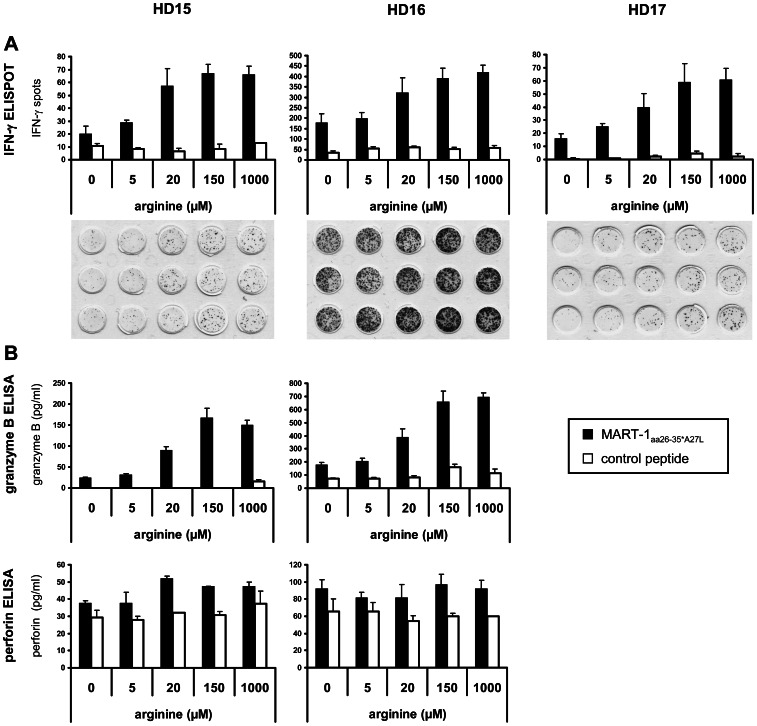
Tumor antigen specific secretion of IFN-γ and granzyme B by human CD8^+^ T cells is regulated by the extracellular arginine concentration, whereas perforin secretion is independent of arginine. Expanded MART-1_aa26–35*A27L_ specific CD8^+^ T cells of 3 HD were activated with MART-1_aa26–35*A27L_ peptide (black bars) or control peptide (white bars) loaded T2 cells in medium with different concentrations of arginine (0, 5, 20, 150 and 1000 µM) in ELISPOT assays. After 24 h supernatants were harvested to determine granzyme B/perforin release by ELISA and IFN-γ was detected on ELISPOT membranes. **A** Results of ELISPOT assays are shown as mean of triplicates. The corresponding ELISPOT membranes of activation with MART-1_aa26–35*A27L_ peptide loaded T2 cells are shown below the diagrams. **B** Granzyme B/perforin concentration in supernatants of ELISPOT assays of 2 HD determined by ELISA.

To assess the correlation between arginine concentration and T cell suppression, the arginine level in the medium of expanded MART-1_aa26–35*A27L_ antigen specific CD8^+^ T cells activated by peptide loaded T2 cells was modified. T cell responses were subsequently analyzed by IFN-γ ELISPOT assay and granzyme B/perforin ELISA (due to technical problems granzyme B/perforin was not measurable for HD 17, data not shown). In all 3 HD, a significant arginine concentration dependent immunosuppression was found below the physiological arginine concentration of 150 µM (Fig. 4). Compared to stimulation of CD8^+^ T cells with MART-1_aa26–35*A27L_ pulsed T2 cells in 1000 µM arginine, we found a median reduction of IFN-γ spots of 3% ±4% (150 µM, p = 0.302), 23% ±10% (20 µM, p = 0.017), 56% ±3% (5 µM, p<0.001) and 70% ±8% (0 µM, p<0.001), respectively. The reduction of granzyme B release was 0% ±11% (150 µM, p = 0.743), 42% ±2% (20 µM, p = 0.002), 75% ±6% (5 µM, p = 0.003) and 79% ±7% (0 µM, p = 0.004). Surprisingly, we found in 2 out of 2 HD an unimpaired perforin secretion in stimulated MART-1_aa26–35*A27L_ specific CD8^+^ T cells in the absence of arginine. The reduction compared to 1000 µM arginine was 0% ±4% (150 µM, p = 0.423), 1% ±15% (20 µM, p = 0.962), 16% ±7% (5 µM, p = 0.085) and 10% ±15% (0 µM, p = 0.423), respectively.

### Human Antigen Specific CD8^+^ T cell Cytotoxicity is Completely Independent of Arginine

**Figure 5:**
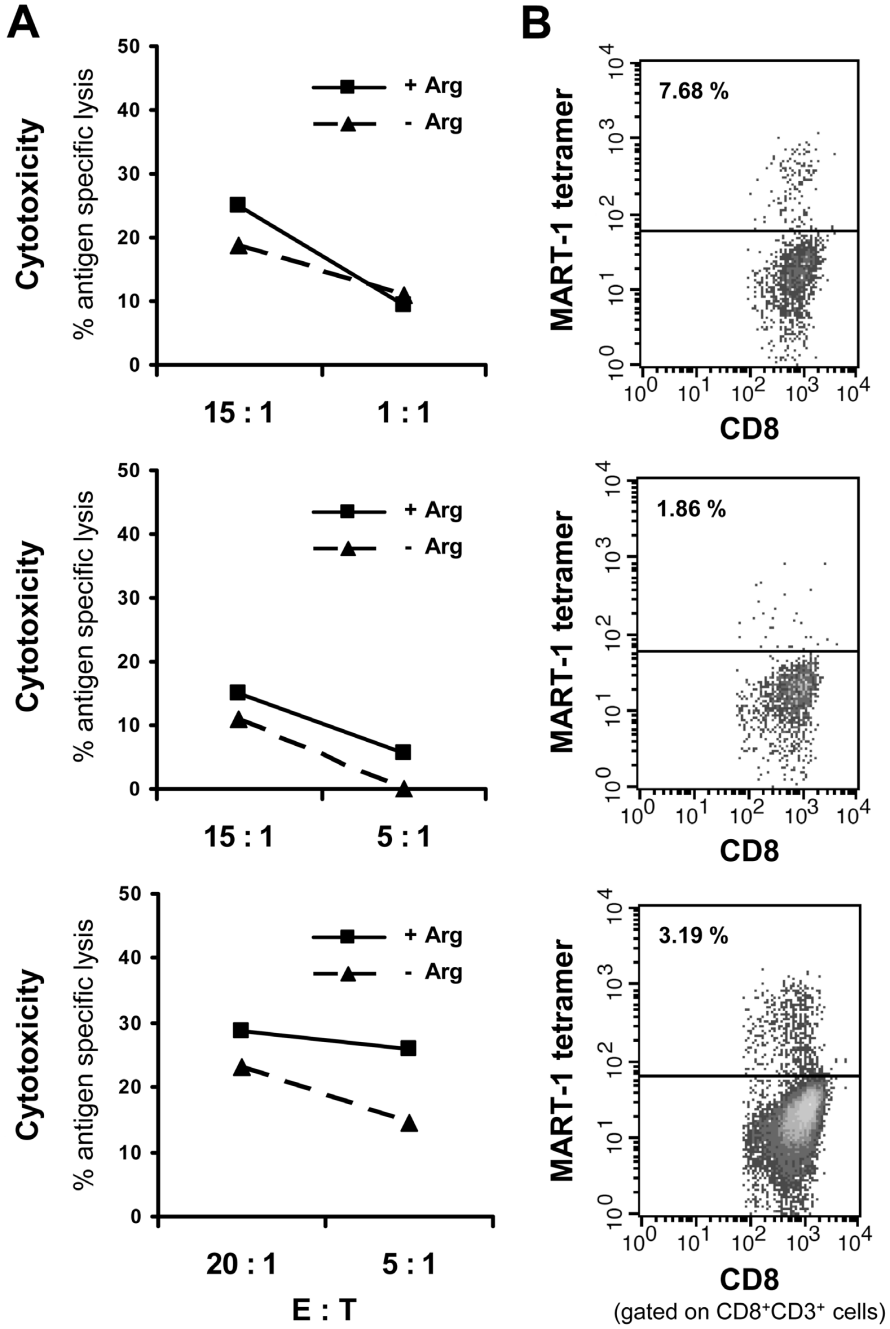
Arginine-independent cytotoxicity of tumor antigen specific T cells. **A** Expanded MART-1_aa26–35*A27L_ specific CD8^+^ T cells of 3 HD were preincubated for 24 h in the presence (+Arg, 1000 µM) or absence (−Arg) of arginine and subsequently stimulated with MART-1_aa26–35*A27L_ peptide or control (irrelevant) peptide pulsed T2 cells for 4 h in a [^51^Cr]-Chromium release assay. Specific lysis of peptide loaded T2 cells upon incubation with MART-1_aa26–35*A27L_ specific CD8^+^ T cells in various E:T ratios (1∶1–20∶1) with/without arginine was determined in duplicates. Shown is the antigen specific lysis in % which is the specific lysis investigated with MART-1_aa26–35*A27L_ loaded T2 cells minus specific lysis with control peptide loaded T2 cells. **B** Quantitative determination of antigen specific T cells was performed by MART-1_aa26–35*A27L_ tetramer analysis and is displayed as % of CD8^+^CD3^+^ T cells (analyzed before separation into the +Arg/−Arg groups) together with the results for the [^51^Cr]-Chromium release assay for each individual experiment.

After demonstrating in the MART-1 antigen specific system that arginine deprivation leads to a suppression of T cell IFN-γ synthesis comparable to the published reports on polyclonally activated T cells, we analyzed the cytotoxic capacity of MART-1_aa26–35*A27L_ specific CD8^+^ T cells. For this cause antigen specific T cells were expanded with peptide loaded DC for 28 days including weekly restimulations with peptide loaded T2 cells. To mimic the *in vivo* situation more closely and to provide time for potential metabolic adaptation we preincubated the CD8^+^ cells in the respective medium (+/− arginine) for 24 h. Figure 5 displays the results of 3 specific [^51^Cr]-Chromium release assays with MART-1_aa26–35*A27L_ specific CD8^+^ T cells from HD, in various E:T ratios ranging from 1∶1 to 20∶1. Shown is the antigen specific lysis obtained in the presence or absence of arginine. Antigen specific lysis was calculated by subtracting specific lysis of T2 cells loaded with control peptide from specific lysis of MART-1_aa26–35*A27L_ loaded ones (Fig. 5A). Quantitative determination of antigen specific T cells was performed by MART-1_aa26–35*A27L_ tetramer analysis and is displayed together with the results of the [^51^Cr]-Chromium release assay for each individual experiment (Fig. 5B). In contrast to the suppression of T cell IFN-γ/granzyme B secretion, but consistent with unimpaired perforin secretion (Fig. 4B) we found largely unimpaired T cell cytotoxicity in the absence of arginine. The median reduction in specific lysis compared to stimulation with arginine was 9% ±7% (high E:T ratio 15∶1/20∶1, p = 0.153) and 13% ±15% (low E:T ratio 5∶1/1∶1, p = 0.206).

### Impact of Arginine Depletion on Proliferation, Cytokine Secretion and Cytotoxic Effector Function of CMV Antigen Specific CD8^+^ T cells

**Figure 6:**
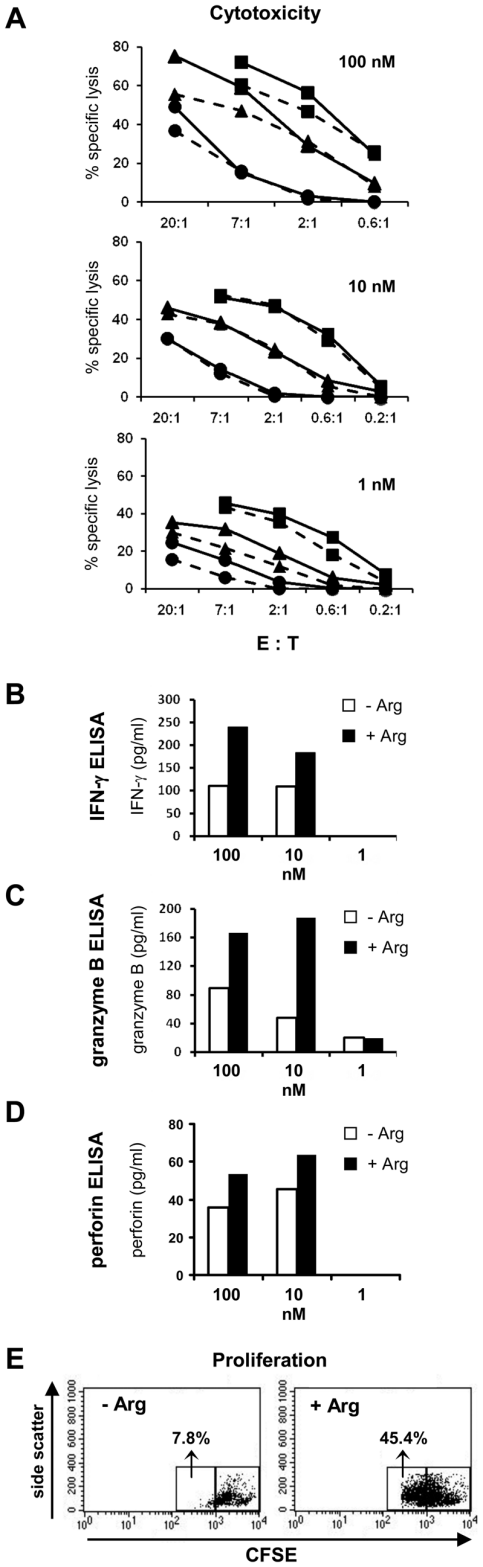
Impact of arginine depletion on cytotoxic capacity, cytokine secretion and proliferation of human CD8^+^ CMV antigen specific T cells expanded from the natural repertoire. Expanded CMV pp65_aa495–503_ specific T cells of 2 HD (•,▴) and a CMV pp65_aa495–503_ specific T cell clone (▪) were activated with pp65_aa495–503_ peptide pulsed K562-A2 cells. **A** Antigen specific tumor cell cytotoxicity was analyzed by [^51^Cr]-Chromium release assay at various E:T ratios and peptide concentrations (100 nM, 10 nM, 1 nM) in the presence (–––) or absence (- - -) of arginine. In parallel, expanded CMV pp65_aa495–503_ specific T cells were incubated with pp65_aa495–503_ peptide pulsed K562-A2 target cells for 48 h (**B–D**) or 120 h (**E**) at an E:T ratio of 20∶1. Supernatant was harvested and concentrations of IFN-γ (**B**), granzyme B (**C**) and perforin (**D**) were determined by ELISA. Data in **A–D** are representative of 3 different experiments with a total of 5 independent HD. **E** Reduced proliferation of CMV pp65_aa495–503_ specific T cells in the absence (−Arg) compared to presence (+Arg, 1000 µM) of arginine, as determined by CFSE staining, gated on all CD3^+^ T cells within the assay mixture. One representative experiment (total: 3) is shown in (E).

To reassess the surprising dichotomous regulation of suppressed (IFN-γ/granzyme B secretion) versus preserved (cytotoxicity) effector functions in a different experimental setting, we used our well-established model of CMV peptide pp65_aa495–503_ specific T cell mediated cytotoxicity. In this system, the frequency of expanded peptide specific T cells is much higher (on average >95% tetramer-positive T cells within the CD8^+^ population) than in the MART-1_aa26–35*A27L_ system. In a first set of experiments, we expanded T cells from the peripheral blood of HD by *in vitro* restimulation. We used these expanded CD8^+^ CMV reactive T cell lines as well as a long-term CMV pp65_aa495–503_ specific T cell clone (clone p21) for *in vitro* cytotoxicity assays at different E:T ratios (with K562-A2 tumor cells as targets) and different pp65_aa495–503_ peptide concentrations either in the presence or absence of arginine. Again T cells were preincubated for 18 h in the respective cell culture media before they were used for the experimental assays. Confirming the data from the MART-1_aa26–35*A27L_ antigen-specific system, depletion of arginine did not significantly inhibit CD8^+^ T cell-mediated cytotoxicity at all tested E:T ratios and peptide concentrations (p>0.05 for all comparisons between arginine-containing (+Arg; 1000 µM arginine) and arginine-deficient media (−Arg); Fig. 6A), whereas secretion of IFN-γ (peptide concentration 100 nM: 332 pg/ml ±225 pg/ml in +Arg medium versus 152 pg/ml ±92 pg/ml in –Arg medium, p = 0.001; peptide concentration 10 nM: 112 pg/ml ±77 pg/ml in +Arg medium versus 65 pg/ml ±46 pg/ml in –Arg medium, p = 0.017; Fig. 6B) and granzyme B (peptide concentration 100 nM: 307 pg/ml ±75 pg/ml in +Arg medium versus 153 pg/ml ±54 pg/ml in –Arg medium, p = 0.01; peptide concentration 10 nM: 269 pg/ml ±91 pg/ml in +Arg medium versus 127 pg/ml ±27 pg/ml in –Arg medium, p = 0.0114; Fig. 6C) was significantly reduced in arginine free medium at both 100 nM and 10 nM CMV pp65_aa495–503_ peptide concentrations (above values are mean ± SD of 3 different experiments with a total of 5 independent HD). In contrast to the suppressed IFN-γ and granzyme B synthesis we again found unimpaired perforin secretion in the absence of arginine also in the CMV pp65_aa495–503_ peptide system (Fig. 6D), recapitulating the findings in the MART-1 experiments (see Fig. 4B). In summary, we measured 35 pg/ml ±23 pg/ml in +Arg medium versus 34 pg/ml ±10 pg/ml in –Arg medium, p = 0.865 for 100 nM peptide concentration and 54 pg/ml ±13 pg/ml in +Arg medium versus 47 pg/ml ±8 pg/ml in –Arg medium, p = 0.4075 for 10 nM peptide concentration; Fig. 6D). Finally, antigen specific proliferation of CMV reactive CD8^+^ T cells was again found to be strongly impaired by arginine depletion (47% ±9% of T cells in proliferation gate in +Arg medium versus 23% ±19% of T cells in proliferation gate in –Arg medium, p = 0.029; mean ± SD of 3 independent experiments; representative experiment in Fig. 6E).

### Effects of Arginine Depletion on Retrovirally Transduced T cells

**Figure 7:**
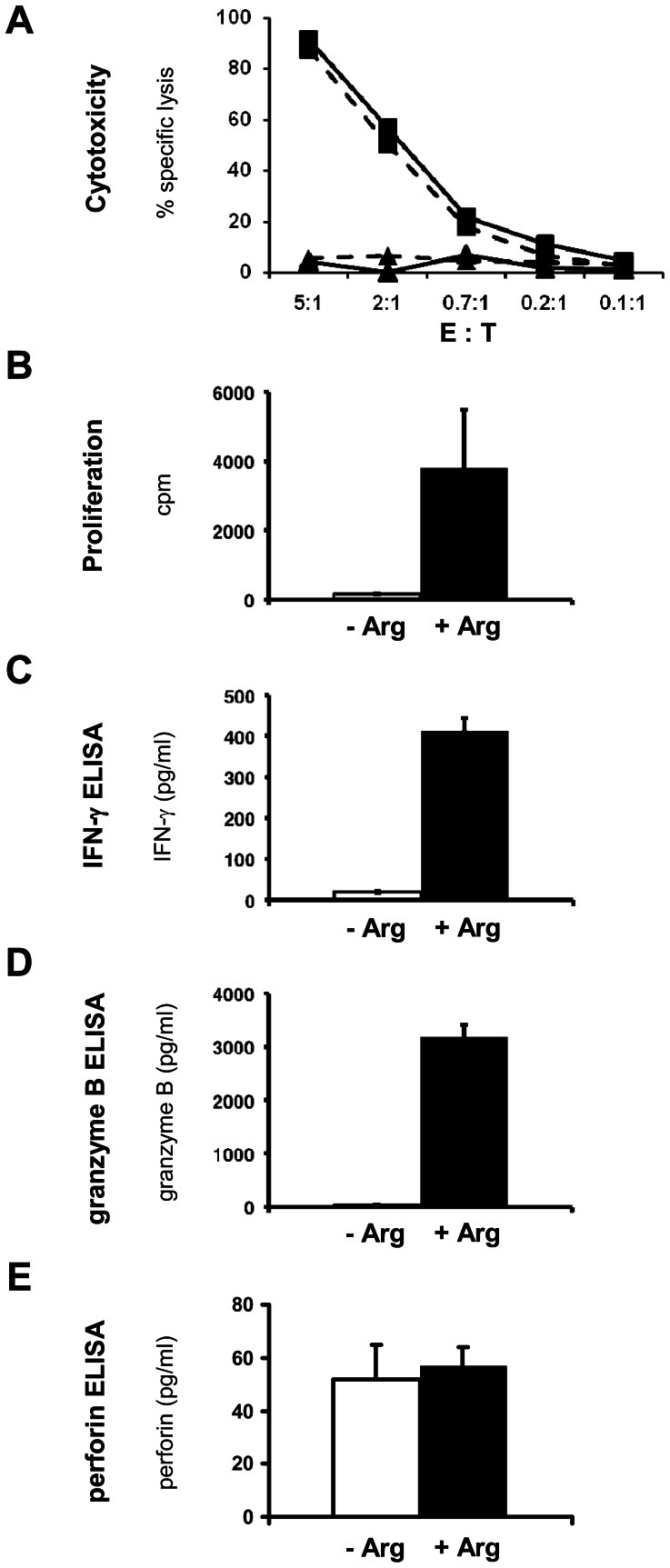
Unimpaired cytotoxicity and perforin secretion but severely suppressed proliferation and effector molecule secretion of retrovirally transduced CMV specific human CD8^+^ T cells. Human primary CD8^+^ T cells retrovirally transduced with a TCR specific for the CMV peptide pp65_aa495–503_ (▪) or mock transduced T cells (▴) were activated with K562-A2 cells pulsed with the cognate pp65_aa495–503_ peptide (100 nM) in the presence (–––) or absence (- - -) of arginine at the indicated E:T ratios (incubation time 4 h). T cell mediated, antigen specific tumor cell cytotoxicity was assessed by [^51^Cr]-Chromium release assay (**A**). In parallel, identical T cell-K562-A2 co-cultivation assays were set up at an E:T ratio of 20∶1 for 48 h (**B–E**). Antigen specific T cell proliferation was assessed by [^3^H]thymidine incorporation for additional 16 h (**B**) while IFN-γ (**C**), granzyme B (**D**) and perforin (**E**) concentration were measured in the 48 h supernatant by ELISA. −Arg: no arginine; +Arg: 1000 µM arginine. Data in **A–E** are representative of 2 different experiments with a total of 4 different TCR constructs and 3 independent HD.

Finally, we addressed the question, if T cells that were retrovirally transduced with a defined TCR might be able to evade the suppression of antigen specific proliferation and cytokine secretion in the context of unimpaired cytotoxicity under arginine deficiency. This experimental approach mirrors the situation of genetically engineered T cells for adoptive cellular immunotherapy. We therefore transduced T cells retrovirally with a CMV pp65_aa495–503_ specific TCR and used these cells for *in vitro* analysis of antigen specific cytotoxicity, proliferation and cytokine secretion. Summarizing two different experiments with a total of four different TCR constructs and three independent HD, the following results were seen: again, CD8^+^ T cell cytotoxicity was completely unaffected in the absence of arginine (no significant differences of specific lysis at all tested E:T ratios between Arg+ (1000 µM) and Arg− (0 µM) condition; Fig. 7A), while T cell proliferation (2133 cpm ±1312 cpm in +Arg medium versus 166 cpm ±145 cpm in –Arg medium, p = 0.0008; Fig. 7B) as well as the secretion of IFN-γ (214 pg/ml ±177 pg/ml in +Arg medium versus 88 pg/ml ±106 pg/ml in –Arg medium, p = 0.008; Fig. 7C) and granzyme B (1535 pg/ml ±1169 pg/ml in +Arg medium versus 349 pg/ml ±162 pg/ml in –Arg medium, p = 0.017; Fig. 7D) were strongly and significantly decreased by arginine depletion. Again, perforin concentrations in the respective supernatants in +Arg medium (60 pg/ml ±16 pg/ml) compared with –Arg medium (48 pg/ml ±11 pg/ml) were not significantly different (p = 0.153).

## Discussion

The adaptive immune response is controlled by the regulated availability of specific amino acids [30]. Our study highlights the following two novel aspects: (i) human T cell functions were analyzed in an antigen specific context (and not upon polyclonal stimulation) and suppression of IFN-γ secretion, granzyme B mobilization and proliferation were recapitulated; (ii) we show that important human T cell functions – chemotaxis, calcium signaling, cytotoxicity – are completely preserved in the absence of arginine.

Peptide specific T cell activation in the context of MDSC-induced arginine depletion was already analyzed in several *murine* models. Murine MDSC from tumor bearing mice can inhibit the OVA peptide antigen specific production of IFN-γ by CD8^+^ T cells via oxidative stress [31], [32] or influenza hemagglutinin [31] or OVA peptide specific [33] T cell proliferation via arginase-mediated arginine depletion. Inhibition of prostate carcinoma specific, tumor infiltrating effector lymphocytes could be reversed and CD8^+^ cytotoxicity and tumor cell apoptosis increased upon arginase and NOS inhibition in a murine tumor model [14]. Also, several murine models exist that demonstrate MDSC- or arginase-mediated inhibition of T cell priming. Expansion of naïve transgenic OVA specific CD4^+^ T cells [34] and hemagglutinin specific CD8^+^ T cells [34] is inhibited by murine tumor-induced MDSC [34] or regulatory DC via arginase [35]. Also, in the murine NeuT tumor model, naïve CD8^+^ T cell priming and antitumoral cytotoxicity is inhibited by arginase-expressing tumor infiltrating DC [36].

In humans, various studies have demonstrated the inhibition of polyclonal or alloreactive T cell stimulation via depletion of arginine. Human immature myeloid cells from advanced stage cancer patients inhibit allogeneic or tetanus toxoid specific T cell responses [37], while CD14^+^ myeloid suppressor cells in melanoma patients inhibit polyclonally activated IFN-γ secretion and proliferation of human T cells [38]. Inhibition of the human MDSC effector mechanisms iNOS and arginase restores T cell proliferation after polyclonal activation via anti-CD3/CD28-coated beads [12] or PMA/PHA [14]. Our current study extends these analyses by demonstrating the impact of arginine depletion on peptide specific human T cell activation in two different antigen systems.

Our findings of unimpaired T cell chemotaxis, calcium signaling, perforin secretion and cytotoxicity extend our recent observation that certain cytokines (IL-2, IL-6 and IL-8) can be synthesized by activated human T cells in an arginine-independent way in contrast to severely suppressed cytokines like IFN-γ, IL-10 or TNF-β [10]. In earlier analysis we could already show that e.g. CD69 upregulation on T cells is even enhanced upon activation in the absence of arginine [9]. The unimpaired calcium flux complements our recent observation that another T cell signaling pathway, namely PI3K activity measured as Akt phosphorylation, is also uncompromised and even hyperactive in the absence of arginine upon T cell stimulation via TCR while e.g. ERK activity is suppressed [10].

It remains to be analyzed how human T cells can preserve their cytotoxic effector function even though arginine deficiency leads to a reduction in granzyme B mobilization (Fig. 4B, 6C). We can exclude a participation of the CD95/CD95L system since our K562-A2 target cells do not express CD95 (data not shown). Also, a participation of TRAIL is rather unlikely, since we did not observe TRAIL expression on the T cells within the 4 h time period of the cytotoxicity assay (data not shown). We saw arginine-independent perforin secretion in the three different antigen-specific T cell activation systems and this might be involved in the unimpaired T cell cytotoxicity in the context of arginine deficiency. Since also other proteins like e.g. granulysin might contribute to this phenomenon, further studies are needed to clarify our novel findings.

We demonstrated recently that cofilin dephosphorylation and actin reorganization are impaired upon activation of human primary T cells in the absence of arginine [10]. Since SDF-1α induced T cell chemotaxis also requires actin reorganization [39] and hyperphosphorylation of cofilin can interfere with SDF-1α induced human T cell migration [40] we would have anticipated an impaired chemotactic T cell movement in arginine-free medium. This is clearly not the case (Fig. 1A) and might be explained by our previous observation that migration of human T cells depends on cofilin-mediated actin reorganization only in the 3D setting (e.g. in vitro matrigel migration) but not (as in our experiments) in 2D circumstances [41]. Further studies are needed to address the redundant signaling pathways in human T cells that allow sufficient actin reorganization for uncompromised chemotaxis in the absence of arginine.

Human MDSC induced T cell immunosuppression clearly differs with respect to the prevailing MDSC-associated immunosuppressive mechanism. While our data show that arginine depletion has no negative impact on CD8^+^ T cell mediated cytotoxicity, others reported that peptide specific IFN-γ secretion and cytolytic activity as well as the tetanus-toxoid specific proliferation of MART-1 tumor antigen specific T cells can be inhibited by oxidative stress induced by MDSC of renal cell carcinoma patients [32]. In contrast to our study, in which we analyzed activation of freshly generated MART-1 tumor antigen specific T cells, the authors used a long-term MART-1 specific T cell clone, which does not necessarily reflect the situation *in vivo* of *de novo* vaccinated patients. Furthermore, tumor-derived lactic acid, like arginine depletion a powerful immunosuppressive effector mechanism, also shows some degree of split T cell inhibition: while T cell proliferation and cytokine secretion are severely suppressed, T cell cytotoxicity is decreased to 50% [42]. All these studies clearly complement each other in their analysis of different tumor immune escape mechanisms and their impact on tumor antigen specific CD8^+^ T cells.

While we demonstrate unimpaired human CD8^+^ T cell cytotoxicity even in the complete absence of arginine, long term immunological tumor control supposedly depends on effective production of various effector cytokines and proapoptotic factors (e.g. granzyme B, perforin) as well as antitumoral T cell expansion and differentiation. We therefore hypothesize that different tumor-induced immunosuppressive effector pathways synergize and eventually lead to fundamental T cell paralysis within the tumor microenvironment.

In summary, we have demonstrated an unexpected selective regulation of human T cell effector functions in the absence of arginine: While antigen specific proliferation and secretion of IFN-γ and granzyme B are strongly suppressed, T cells completely preserve their cytotoxic and chemotactic functions. Our data highlight the complexity of tumor-induced immune escape mechanisms and emphasize the need for counterregulatory immunotherapeutic strategies to enhance the endogenous or therapeutically-induced anti-tumor immune response.
